# Arthroscopic resection of wrist ganglia

**DOI:** 10.1186/1753-6561-9-S3-A31

**Published:** 2015-05-19

**Authors:** Christophe Mathoulin

**Affiliations:** 1 Institut de la Main, 75016, Paris, France

## Introduction

The purpose of this study is to analyse the rate of recurrences of the dorsal ganglion resected by trans-cystic arthroscopic technique. All patients were operated on by a single surgeon with a minimum follow-up of 2 years.

## Material and methods

We report a series of 114 patients with dorsal ganglia (87 women and 27 men). The average age was 33.2 years (range:12 to 63 years). All the patients had had an unsuccessful preliminary treatment. Although a minority of patients (32 cases) complained of pain, aesthetic motivation comprised the majority of cases (82 patients).

All the patients were operated under local-regional anaesthesia on an outpatient basis. The shaver was placed directly in trans-cystic, mostly into the midcarpal joint, to perform the capsulectomy and resection of the wall of cyst. The patients were allowed to mobilise their wrist freely immediately post-operatively.

## Results

Our average follow-up was 42.3 months (between 24 and 84 months). The functional clinical data (mobility, pain and strength) were improved in a statistically significant way. We had 14 recurrences (12 %) which appeared after an average periodof 16.8 months (between 2 and 35 months). The cyst was again removed by wrist arthroscopy in 11 cases at the request of the patients without any particular problem. We had no other complications

## Conclusion

The arthroscopic resection of dorsal ganglia of the wrist is a technique which allows a satisfactory resection of the cyst and the adjacent articular capsule. The rate of recurrence appears significant initially but it is within comparable levels to the best series of classical open techniques. The long follow-up of this series addresses the notion of true recurrence or return of a new cyst. Nevertheless the rate of satisfaction of the patients is very high particularly because there are no requirements for cutaneous suture or immobilization of the wrist.

